# Analysis of a model for bacteriophage infections and bacteria defense: a synergetics perspective

**DOI:** 10.3389/fnetp.2025.1657313

**Published:** 2025-09-19

**Authors:** T. D. Frank

**Affiliations:** ^1^ Department of Psychological Sciences, University of Connecticut, Storrs, CT, United States; ^2^ Department of Physics, University of Connecticut, Storrs, CT, United States

**Keywords:** network physiology, bacteriophages, infection dynamics, order parameters, synergetics

## Abstract

A model for bacteriophage infections and bacteria defense is analyzed using the concepts of synergetics. The model order parameter is determined and the corresponding amplitude equations are derived. Within this framework it is shown how the order parameter defines a multi-species building block that captures the organization of infection outbreaks and the initial defense reaction and how the order parameter amplitude determines the corresponding temporal characteristics. Two approximative models with different domains of application are derived as well. In doing so, a supplementary perspective of bacteriophage infections that provides insights beyond the classical state space perspective is provided.

## 1 Introduction

In general, epidemiological systems are given by complex networks of interacting populations of different species (Pastor-Satorras et al., 1998). While the isolated populations typically exhibit a relatively simple dynamics, a challenge in the field of network physiology is to understand how the interactions between the different types of species shape the overall network dynamics. In this context, an important first step is to consider mean field approximations in terms of ODE and coupled differential equation models (Pastor-Satorras et al., 1998; Granger et al., 2024), which due to their relative simplicity frequently allow for analytical solution methods. For virus infections the ODE three-species TIV model captures the basic dynamics of interacting target cells, infected cells, and virus particles (Nowak and May 2000). Likewise, in the context of bacteriophage infections, we are dealing with target bacteria, infected bacteria, and bacteriophages–the latter act as viruses. Studying bacterial infections and the role of bacteriophages is an important task (Bloch and Wegrzyn, 2024; Geng et al., 2022; Zborowsky et al., 2025) and is an indispensable step when attempting to use phages in modern medicine to cure certain diseases in humans (Li et al., 2021; Zborowsky et al., 2025). To this end, both simplified and generalized three-species models have been studied in the literature (Li et al., 2021; Weitz et al., 2005; Zborowsky et al., 2025). In particular, as part of their comprehensive study, Skanata and Kussell (2021) considered bacteria that can exist as resistant and non-resistant phenotypes with respect to a given invading phage. Increasing the concentration of resistant phenotypes is a defense mechanism against phage attack because this mechanism decreases the effective contact rate between phage and non-resistant bacteria such that under appropriate conditions the infection dies out. While the dynamics systems perspective, in general, is an indispensable tool to analyze bacteriophage infection models, little attention has been paid to utilize the more specific dynamical systems concepts of synergetics (Haken, 2004; Hutt and Haken, 2020; Uhl, 1999; Wunner and Pelster, 2016) in this regard. However, in the wake of the COVID-19 pandemic it has been shown that synergetics can supplement existing dynamic systems approaches to understand epidemiological and virus dynamic models (Frank, 2022). In this Brief Report a parsimonious four-species model for bacteriophage infection and bacteria defense will be considered that involves resistant phenotypes as in Skanata and Kussell (2021). The relative simplicity of the model will allow for an analytical approach. The aim of the study is to derive explicitly the order parameter of the model and to show how it determines the initial organization of a phage attack and the corresponding defense reaction. Moreover, the aim is to derive the amplitude equations that determine the evolution of the system along the order parameter and the remaining directions. Two approximative models for the system dynamics in this context will be derived as well. An exemplary simulation will illustrate some of the analytical results.

## 2 Materials and methods

As reviewed above, our starting point is the three-species model that involves susceptible (S) and infected (I) bacteria and phage load (P) . In line with the TIV model of virus dynamics (Frank, 2022) the model equations read

$$\frac{\text{d}}{\text{d}t}S=-k_{0}\left(P\right)\;S+\mu S\left(1-\frac{S}{K}\right),\;\frac{\text{d}}{\text{d}t}I=k_{0}\left(P\right)\;S-k_{1}I,\frac{\text{d}}{\text{d}t}P=qI-k_{2}P,\;$$ (1)

with $k_{1},k_{2},q>0$ , where t denotes time, $k_{0}$ describes the P -dependent transition rate of S→I transitions, $k_{1}$ and $k_{2}$ denote the decay rates of infected cells and phages, respectively, and q describes the production rate of phages per infected bacteria. The evolution equation for S involves a logistic growth term with the growth rate μ and the capacity K>0 . Below we will consider both the case μ=0 when the growth term can be neglected and the more general case μ>0 . By comparing these two cases, we will see that the bacterial growth dynamics actually has no effect on the initial outbreaks dynamics captured by the order parameter. Therefore, the growth term may be neglected when (i) changes in S are primarily due to S→I transitions capture by the $k_{0}S$ -term or (ii) the focus is on the initial phase of the outbreak dynamics. The transition rate $k_{0}\left(P\right)$ in Equation 1 depends on the infecting species like $k_{0}=\beta_{0}P$ , where $\beta_{0}>0$ denotes the effective contact rate (Frank, 2022). In order to take the active defense mechanism mentioned in the introduction into account, the model (1) was modified in two ways. First, it was assumed that when infected bacteria I emerge in the bacteria population then resistant bacteria mutations (R) are grown like

$$\frac{\text{d}}{\text{d}t}R=\alpha I\left(1-\frac{R}{R_{m}}\right),$$ (2)

where α≥0 and $R_{m}>0$ denote the growth rate and the maximal concentration of resistant bacteria, respectively. From a mechanistic point of view, Equation 2 captures the adaptive defense mechanism of bacteria via the so-called CRISPR system (Abedon, 2012; Skanata and Kussell, 2021). The CRISPR system allows bacteria to memorize attacking phages such that they become immune against future attacks. In doing so, in the presence of invading phages phage-resistant bacteria emerge. Second, in general, there are several mechanism with which resistant bacteria R may slow down or stop a bacteriophage infection (Skanata and Kussell, 2021). Again, for sake of brevity, only the effect of R on the S→I transition rate was considered. By doing so, the transition rate $k_{0}$ becomes a function of R and P and reads (Skanata and Kussell, 2021)

$$k_{0}\left(P,R\right)=\frac{\beta_{0}}{1+\gamma R}P,$$ (3)

where γ≥0 measures the effectivity of the active defense mechanism. Basically, Equation 3 states that the presence of resistant cells lowers the chance of an effective contact between phages and susceptible bacteria, such that the R -dependent effective contact rate reads $\beta =\beta_{0}/\left(1+\gamma R\right)$ .

Taking a synergetics perspective (Haken, 2004; Frank, 2022), for the model (1–3) the order parameter and the amplitude equations were derived. To this end, using the state vector X=(S,I,P,R) , bacteriophage infections were considered that start close to an initial fixed point $X_{st,0}=\left(S_{st,0},I_{st,0},P_{st,0},R_{st,0}\right)$ (see Results and Discussions section) of the model. Subsequently, with the help of the eigenvectors $v_{j}$ obtained from a linear stability analysis the amplitudes $A_{j}$ were implicitly defined by

$$X=X_{st,0}+\overset{4}{\underset{j=1}{\sum }}A_{j}v_{j}.$$ (4)

By constructing a bi-orthogonal basis (Haken, 2004; Frank, 2022) spanned by the vectors $w_{j}$ with $w_{i}v_{k}=\delta_{ik}$ (Kronecker symbol), the amplitudes were explicitly expressed like

$$A_{j}=w_{j}u=w_{j}\left(X-X_{st,0}\right),$$ (5)

where u denotes the difference vector $u=X-X_{st,0}$ . From the model Equations 1–3 and Equation 5, eventually the model amplitude equations of the form

$$\frac{\text{d}}{\text{d}t}A_{j}=\lambda_{j}A_{j}+N_{j}\left(A\right)$$ (6)

were derived for j=1,2,3,4 with A constituting the amplitude vector $A=\left(A_{1},A_{2},A_{3},A_{4}\right)$ . In Equation 6 $\lambda_{j}$ denote the eigenvalues of the system and $N_{j}$ are nonlinear functions in the amplitudes. The order parameter and its order parameter amplitude were identified as the eigenvector $v_{j}$ and its amplitude $A_{j}$ corresponding to the potentially positive eigenvalue $\lambda_{j}$ of the model (Haken, 2004; Frank, 2022).

## 3 Results and discussions

### 3.1 Amplitude equation perspective

The fixed-point analysis showed that the model (1–3) exhibits the phage-free fixed points defined by

$$S_{st}\geq 0,\;I_{st}=0,\;P_{st}=0,\;R_{st}\in \left[0,R_{m}\right]$$ (7)

for μ=0 . For μ>0 Equation 7 holds with $S_{st}=K$ . As mentioned in the Methods section, it is assumed that at time t<0 , i.e., before the infection takes place, the system stays in a fixed point (7). The fixed point is referred to as initial fixed point and denoted by $X_{st,0}$ . At time t=0 the bacteria population is infected by phages of concentration P(0)>0 such that the state is shifted out of its fixed point. Consequently, the model describes infection outbreaks that begin with an initial phage infection of P(0)>0 at time t=0 and end in a phage-free state defined by Equation 7 or an endemic state if it exists (see below).

The linear stability analysis at $X_{st,0}$ showed that the model for μ≥0 exhibits the eigenvalues $\lambda_{1}=-\mu$ , $\lambda_{4}=0$ , as well as

$$\lambda_{2,3}=-\frac{k_{1}+k_{2}}{2}\pm \sqrt{\frac{{\left(k_{1}+k_{2}\right)}^{2}}{4}-k_{1}k_{2}+q\beta_{0}f_{st,0}S_{st,0}}$$ (8)

with $f_{st,0}=1/\left[1+\gamma R_{st,0}\right]$ , where the upper (lower) sign holds for $\lambda_{2}$ $\left(\lambda_{3}\right)$ . For μ>0 in Equation 8 and in what follows we must substitute $S_{st,0}=K$ . It can be shown that for arbitrary model parameters $\lambda_{3}<0$ holds. In contrast, $\lambda_{2}$ can assume positive or negative values. In this context, note that using the next-generation method, the basic reproduction number $R_{0}$ of the model (Frank, 2022) can be obtained as $R_{0}=q\beta_{0}f_{st,0}S_{st,0}/\left(k_{1}k_{2}\right)$ . Case I is defined by $q\beta_{0}f_{st,0}S_{st,0}<k_{1}k_{2}\Leftrightarrow \lambda_{2}<0$ , which is equivalent to $R_{0}<1$ , such that the fixed point $X_{st,}$ is a neutrally stable/asymptotically stable fixed point for μ=0 and μ>0 , respectively. There is no infection outbreak. Tn contrast case II is characterized by $q\beta_{0}f_{st,0}S_{st,0}>k_{1}k_{2}\;\Leftrightarrow \;\lambda_{2}>0$ , which is tantamount to say that $R_{0}>1$ holds. The fixed point is unstable. The infection dynamics describes an infection outbreak. The inequality means that the infection outbreak scenario, i.e., case II, occurs when the system parameters q and $\beta_{0}$ are relatively large, the initial value $S_{st,0}$ is relatively large, while the initial concentration $R_{st,0}$ is relatively small. For μ=0 the model exhibits only phage-free fixed points. A detailed calculation shows that for μ>0 an endemic fixed point exists if the defense mechanism via the R dynamics cannot stabilized the phage-free fixed point with $S_{st,0}=K$ . Mathematically speaking, if $\lambda_{2}>0$ holds for $S_{st,0}=K,R_{st,0}=R_{m}$ , which is equivalent to say that $k_{1}k_{2}<q\beta_{0}f_{st,R\left(max\right)}K$ holds (where $f_{st,R\left(max\right)}=1/\left(1+\gamma R_{m}\right)$ ), then an endemic fixed point with $I_{st}\in \left(0,K\right)$ and $P_{st}>0$ exists. Having said that since the objective of the study is examine initial infection outbreaks from the phage-free state, we will not dwell on the endemic state.

The linear stability analysis of the phage-free fixed point produced the eigenvectors $v_{1}=\left(1,0,0,0\right)$ and $v_{4}=\left(0,0,0,1\right)$ associated with the zero eigenvalues $\lambda_{1}$ and $\lambda_{4}$ . For j=2,3 the eigenvectors read as shown in Equation 9

$$v_{j}=\frac{1}{Z_{j}}\left(\begin{array}{c} -F_{0}\left(\lambda_{j}+k_{1}\right)\frac{\lambda_{j}}{\lambda_{j}+\mu }\\ F_{0}\lambda_{j}\\ \left(\lambda_{j}+k_{1}\right)\lambda_{j}\\ F_{0}\alpha \left(1-R_{st,0}/R_{m}\right) \end{array}\right)$$ (9)

with $F_{0}=\beta_{0}f_{st,0}S_{st,0}$ , where $Z_{j}$ is a normalization constant such that $|v_{j}|=1$ . It was found that the bi-orthogonal vectors $w_{2,3}$ of the model exhibit the well-known structure from other epidemiological models (Frank, 2022): $w_{2}=B^{-1}\left(0,v_{3,P},-v_{3,I},0\right)$ and $w_{3}=B^{-1}\left(0,-v_{2,P},v_{2,I},0\right)$ , where $v_{j,I}$ and $v_{j,P}$ denote the I and P coordinates of the eigenvectors $v_{j}$ , respectively. Here $B=v_{2,I}v_{3,P}-v_{2,P}v_{3,I}$ . A detailed calculation showed that $w_{1}$ and $w_{4}$ associated with $v_{1}=\left(1,0,0,0\right)$ and $v_{4}=\left(0,0,0,1\right)$ , respectively, read as shown in Equation 10

$$w_{1}=\frac{1}{\lambda_{3}-\lambda_{2}}\left(\begin{array}{c} \lambda_{3}-\lambda_{2}\\ \left(\lambda_{2}+k_{1}\right)\left(\lambda_{3}+k_{1}\right)\left[\frac{1}{\lambda_{2}+\mu }-\frac{1}{\lambda_{3}+\mu }\right]\\ -F_{0}\left[\frac{\lambda_{2}+k_{1}}{\lambda_{2}+\mu }-\frac{\lambda_{3}+k_{1}}{\lambda_{3}+\mu }\right]\\ 0 \end{array}\right),w_{4}=\frac{1}{\lambda_{2}\lambda_{3}}\left(\begin{array}{c} 0\\ -\alpha^{*}\left(\lambda_{2}+\lambda_{3}+k_{1}\right)\\ \alpha^{*}F_{0}\\ \lambda_{2}\lambda_{3} \end{array}\right)$$ (10)

with $\alpha^{*}=\alpha \left(1-R_{st,0}/R_{m}\right)$ . As in other virus dynamics models (Frank, 2022), the nonlinear functions $N_{j}$ occurring in the amplitude Equation 6 can be expressed as projections of a nonlinear vector-valued function G on the biorthogonal vectors $w_{j}$ like

$$N_{j}\left(A\right)=w_{j}G\left(\delta \left(A\right),I\left(A\right),P\left(A\right),\omega \left(A\right)\right)$$ (11)

with $\omega =R-R_{st,0}$ . That is, δ,I,P,ω are the coordinates of the difference vector u introduced in the Methods section. A detailed calculation showed the results shown in Equations 12, 13 that

$$G=\left(-G_{I}-\frac{\mu }{K}\delta^{2},G_{I},0,G_{R}\right)$$ (12)

and

$$G_{I}=\beta_{0}P\left(\frac{S_{st,0}+\delta }{1+\gamma \left[R_{st,0}+\omega \right]}-\frac{S_{st,0}}{1+\gamma R_{st,0}}\right),\;G_{R}=-\alpha I\frac{\omega }{R_{m}}$$ (13)

As indicated in Equation 11, the variables δ,I,P,ω are expressed in terms of $A_{j}$ . Explicitly, we have $u=\left(\delta ,I,P,\omega \right)=\sum_{j}A_{j}v_{j}$ , see Equation 4. Consequently, the amplitude equations defined by Equation 6 and (11–13) form a closed set of coupled differential equations.

### 3.2 Implications

#### 3.2.1 Order parameter: essential building-block and initial organization

The model exhibits maximally one positive eigenvalue. Consequently, under the case II scenario with $\lambda_{2}>0\left(R_{0}>1\right)$ the system exhibits an order parameter given by $v_{2}$ and the order parameter amplitude $A_{2}$ (Haken, 2004; Frank, 2022). Let us split the state dynamics into two parts $X=X_{\mathrm{out}}+X_{s}$ , where $X_{s}=A_{3}\left(t\right)v_{3}$ for μ=0 and $X_{s}=A_{1}\left(t\right)v_{1}+A_{3}\left(t\right)v_{3}$ for μ>0 describes the dynamics along the stable direction(s) and $X_{\mathrm{out}}$ captures the remaining dynamics. Initially, i.e., for t≈0 , we have

$$X_{\mathrm{out}}\approx K+v_{2}A_{2}\left(0\right)\exp\left(\lambda_{2}t\right)$$ (14)

with K= constant and $K=X_{st,0}+A_{1}\left(0\right)v_{1}+A_{4}\left(0\right)v_{4}=$ for μ=0 , whereas $K=X_{st,0}+A_{4}\left(0\right)v_{4}=$ for μ>0 . Equation 14 describes the dynamics along the unstable direction away from the initial fixed point (i.e., the outwards dynamics). In contrast, $X_{s}$ describes the dynamics towards the unstable direction, i.e., towards the order parameter. Consequently, the order parameter $v_{2}$ describes the emergent organization of the multi-species physiological network and its amplitude $A_{2}$ describes how this organization evolves over time.

Since $X_{s}$ initially decays in magnitude over time, when considering the initial infection dynamics we may neglect its contribution to the state dynamics. If so, then any state change defined by $\Delta X=X\left(t\right)-X_{st,0}$ approximately is given by

$$\Delta X\approx v_{2}\Delta A_{2}\approx v_{2}\left(\exp\left(\lambda_{2}t\right)-1\right).$$ (15)

Equation 15 illustrates again that the order parameter describes the essential building-block that shapes an infection outbreak in the multi-species network under consideration including the defense reaction (see component $v_{2,R}$ ).

#### 3.2.2 Stopping mechanisms

The exponential increase along $v_{2}$ as described by Equation 15 is slowed down and eventually stopped at some point in time. In line with the stability analysis let us assume that δ,I,P,ω are small quantities of the order ϵ . Then $N_{2}$ when interpreting $N_{2}$ as a function of the difference variables can be expanded such that the amplitude equation for $A_{2}$ reads

$$\frac{\text{d}}{\text{d}t}A_{2}=\lambda_{2}A_{2}-\underset{>0}{\underbrace{v_{3,p}\beta_{0}f_{st,0}}}\underset{\geq 0}{\underbrace{\left(\gamma S_{st,0}f_{st,0}P\omega -P\delta \right)}}+O\left(\epsilon^{3}\right).$$ (16)

Note that δ<0 for any t>0 . Consequently, the network physiology produces two mechanisms that slow down the exponential increase of $A_{2}$ : the decay in susceptibles in the presence of phages as measured by the interaction term −Pδ>0 and the increase of the number of resistant bacteria again in the presence of phages as measured by the interaction term Pω>0 . The former mechanism is a passive mechanism that simply states that the exponential infection outbreak slows down due to a decay of the resources (i.e., susceptible bacteria). The latter mechanism is an active mechanism that states that the introduction of resistant bacteria mutations into the bacteria colony has the desired effect of slowing down the phages invasion.

#### 3.2.3 Linear predictor equations

Equation 15 implies that all species initially satisfy linear regression equations of the form as shown in Equation 17

$$X_{i}\approx a_{i,j}+r_{i,j}X_{j},\;r_{ij}=v_{2,i}/v_{2,j}.$$ (17)

Accordingly, any species of the network can be used to predict any other network species (assuming $r_{ij}\neq 0$ for all i,j ). The network components are coupled by linear order parameter links. For example, the bacteriophage population size P may be used to construct regression models like

$$S=a_{S,P}-\frac{F_{0}}{\lambda_{2}}P,\;I=\frac{F_{0}}{\lambda_{2}+k_{1}}P,\;R=a_{R,P}+\frac{\alpha F_{0}}{\left(\lambda_{2}+k_{1}\right)\lambda_{2}}P,$$ (18)

where $a_{X\left(i\right),X\left(j\right)}$ are intercept parameters depending on $X_{st,0}$ . As indicated in Equation 18, $a_{I,P}=0$ because of $I_{st,0}=P_{st,0}=0$ .

#### 3.2.4 Limited impact of bacterial growth term

Clearly, the bacterial growth term may affect the S dynamics. However, it does not affect the order parameter eigenvalue $\lambda_{2}$ and it does not affect the orientation of the order parameter $v_{2}$ in the 3D space (I,P,R) , which is of primary concern. Consequently, the initial outbreak dynamics in the (I,P,R) space as determined by the order parameter dynamics (see Section 3.2.1) is completely unaffected by the bacterial growth term.

### 3.3 2D infected/infectious species dynamics and double exponential dynamics approximation

The dynamics of the infected and infectious species I and P is completely described by the amplitudes $A_{2}$ and $A_{3}$ . The reason for this is that the eigenvectors $v_{1}$ and $v_{4}$ do not exhibit components in the I−P subspace. The mapping from $A_{2}$ and $A_{3}$ to I and P reads

$$\left(\begin{array}{c} I\\ P \end{array}\right)=v_{2}^{\prime }A_{2}+v_{3}^{\prime }A_{3},$$ (19)

where $v_{2}^{\prime }$ and $v_{3}^{\prime }$ denote the projections of $v_{2}$ and $v_{3}$ into the I - P subspace. From Equation 19 it follows that the initial evolution of I and P satisfies a double-exponential function as shown in Equation 20

$$\left(\begin{array}{c} I\\ P \end{array}\right)\approx v_{2}^{\prime }A_{2}\left(0\right)\exp\left(\lambda_{2}t\right)+v_{3}^{\prime }A_{3}\left(0\right)\exp\left(\lambda_{3}t\right),$$ (20)

### 3.4 Scaled model

Using the variable transformations $s=S/S_{st,0}$ , $i=I/S_{st,0}$ , $p=k_{1}P/\left(qS_{st,0}\right)$ , $r=R/R_{m}$ , the model (1–3) becomes

$$\frac{\text{d}}{\text{d}t}s=-k_{0}^{\prime }s+\mu s\left(1-s\right),\;\frac{\text{d}}{\text{d}t}i=k_{0}^{\prime }s-k_{1}i,\frac{\text{d}}{\text{d}t}p=k_{1}i-k_{2}p,\frac{\text{d}}{\text{d}t}r=\alpha^{\prime }I\left(1-r\right)$$ (21)

with

$$k_{0}^{\prime }\left(r,p\right)=\frac{\beta_{0}^{\prime }}{1+\gamma^{\prime }r}p$$ (22)

and $\beta_{0}^{\prime }=\beta_{0}S_{st,0}q/k_{1}$ , $\gamma^{\prime }=\gamma R_{m}$ , and $\alpha^{\prime }=\alpha S_{st,0}/R_{m}$ . Among other things, the scaled model exhibits the following two properties. First, the variables s,i,p,r are dimensionless. Second, the variable transformation P→p turns the phage variable P into a bacteria-like variable (Frank, 2022). That is, just as the model describes that a susceptible bacteria turns into an infected bacteria, the scaled model describes that an infected bacteria turns into a phage unit in a 1:1 manner when expressing phages in the variable p (rather than in P ). Mathematically speaking, from Equation 21 it follows that p increases due to the term $k_{1}I$ at the same rate as the number of infected bacteria decays due to the term $-k_{1}I$ , which means that the model describes the aforementioned 1:1 transition.

### 3.5 Simulation

**FIGURE 1:**
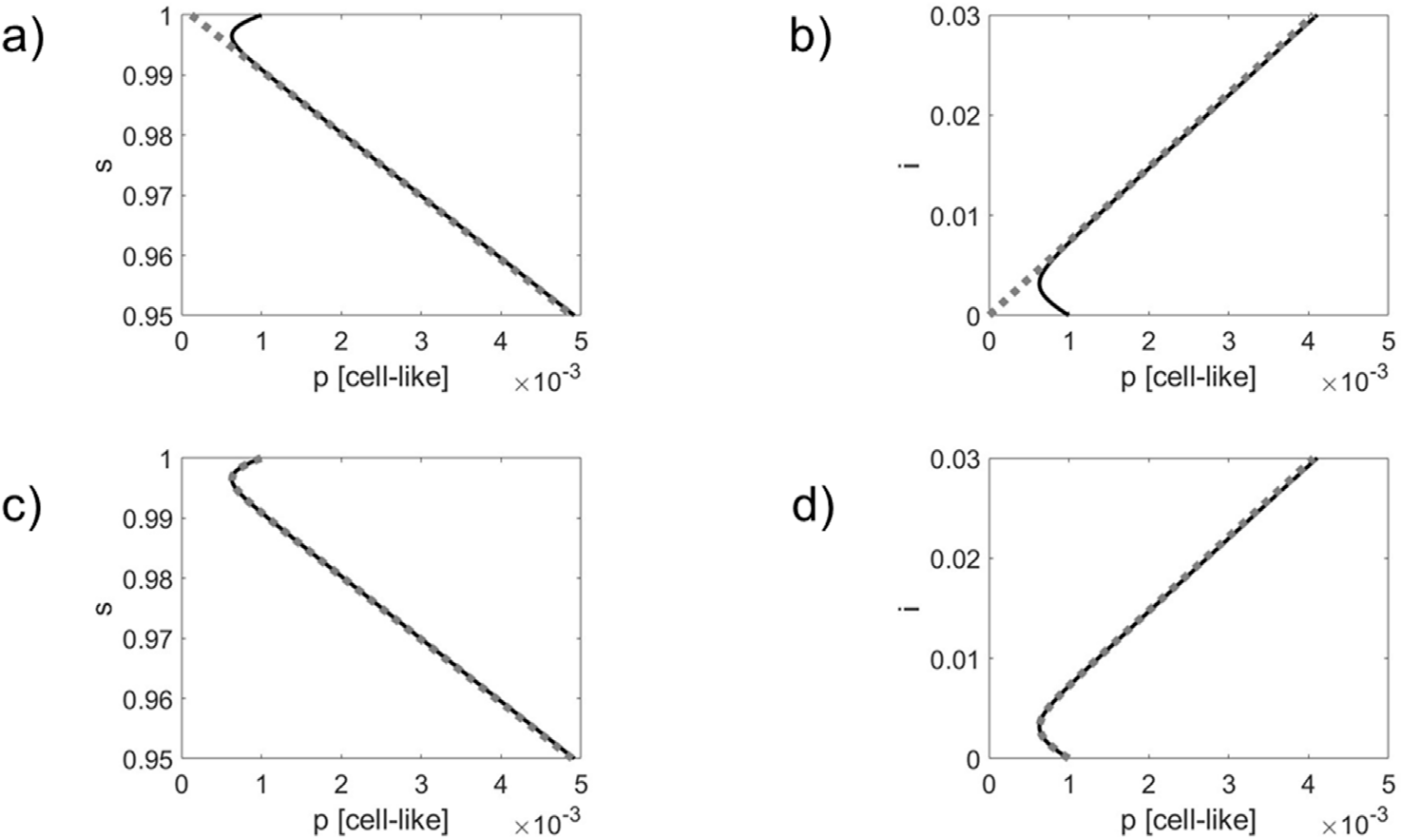
Comparison of simulated infection dynamics (solid black lines) with the order parameter dynamics (panels) **(a,b)** and the double exponential approximation (panels) **(c,d)** shown as gray dotted lines. Phase curves in 2D s−p state spaces (panels **(a,c)** and i−p state spaces (panels) **(b,d)** are shown.

**FIGURE 2:**
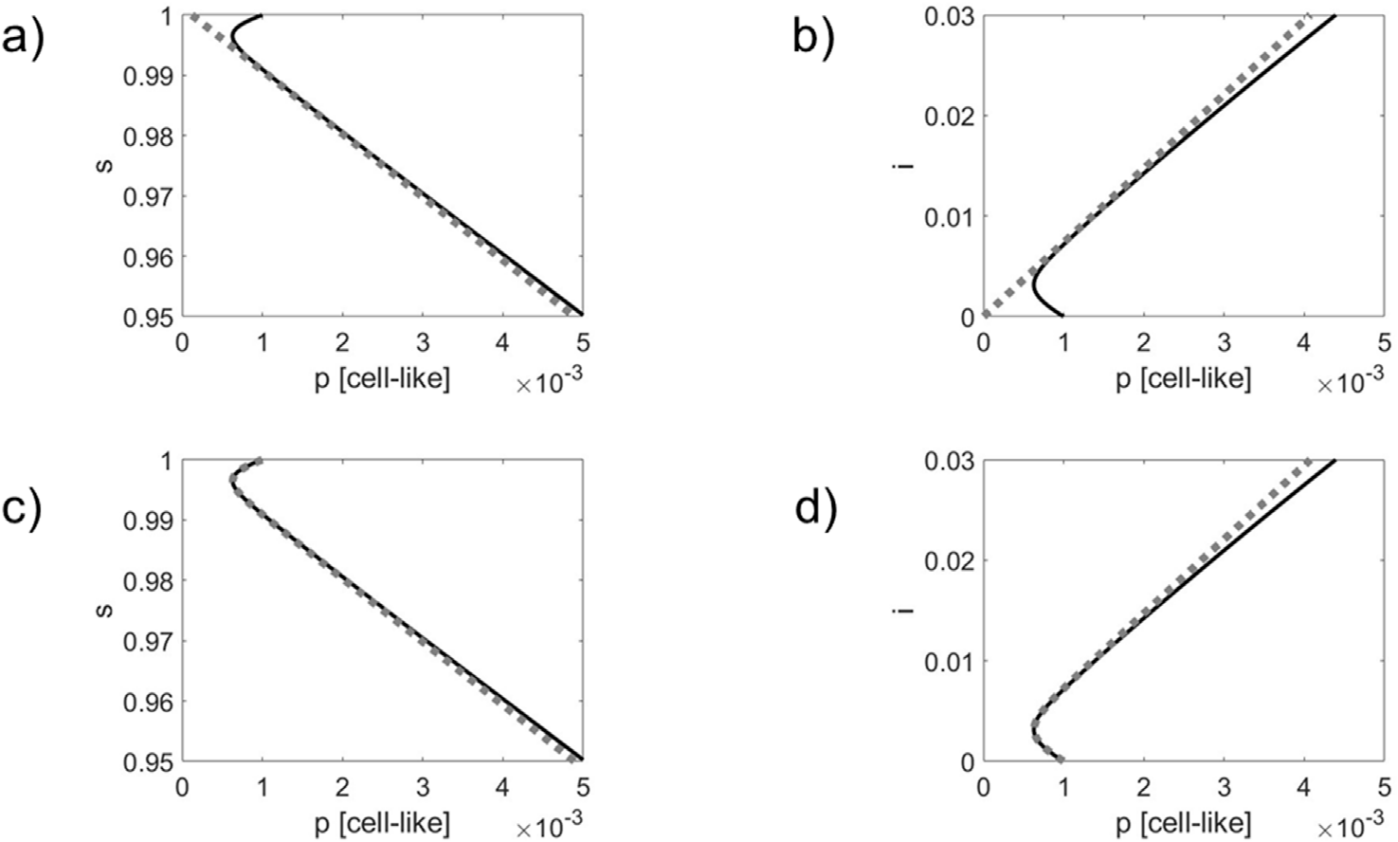
As for Figure 1 but for a simulation that takes the active defense mechanism via resistant bacteria into account. Comparison of simulated infection dynamics (solid black lines) with the order parameter dynamics (panels) **(a,b)** and the double exponential approximation (panels) **(c,d)** shown as gray dotted lines. Phase curves in 2D s – p state spaces (panels **(a,c)** and i – p state spaces (panels) **(b,d)** are shown.

An Euler forward simulation scheme was used to solve Equations 21 and 22. In a first simulation, see Figure 1, only the passive defense mechanism was considered with α=0 and γ=0 . In a second simulation, see Figure 2, the active defense mechanism was taken into account with α=1 and γ=5 . The remaining parameters and initial conditions were $k_{1}=0.2$ /days (Li et al., 2021), $k_{2}=1$ /day, $\beta_{0}^{\prime }=5$ /day, $s_{0,st}=1,i_{st,0}=0,p_{st,0}=0,r_{st,0}=0.01$ , and p(t=0)=0.001 . For sake of brevity, only the most relevant phase curves will be presented and only the first week of the initial outbreak stage will be considered. In this context note that in line with our discussion in Section 3.2.4. In both simulations μ=0 was used.

As can be seen in panels (a) and (b) of Figures 1, 2, the phase curves quickly converge towards the order parameter $v_{2}$ and, subsequently, evolve along $v_{2}$ . By definition, the order parameter $v_{2}$ does not capture the dynamics along the stable direction $v_{3}$ . As can be seen in panels (c) and (d) of Figures 1, 2, the double exponential approximations can describe the transient initial dynamics towards the order parameter (i.e., the dynamics along $v_{3}$ ) as well as the subsequent dynamics along the order parameter $v_{2}$ .

Comparing Figures 1, 2, it can be seen that due to the impact of the phage resistant bacteria the actual dynamics differs from the order parameter dynamics to a greater extent. Likewise, the actual dynamics departs earlier from the double exponential approximations. These observations do not come as a surprise since the active defense mechanism slows down and eventually stops the infection outbreak, see Equation 16. Therefore, the actual dynamics will deviate earlier from the order parameter dynamics, on the one hand, and the double exponential dynamics, on the other hand. Roughly speaking, the active mechanisms weakens the linear order parameter link between the network components.

## 4 Conclusions and limitations

We conclude that under appropriate conditions as specified in the Methods and Simulation sections the order parameter and its amplitude characterize the (self-)organization of a bacteriophage infection and the corresponding bacterial defense. In physics various experiments have been conducted to test specifically predictions of the theory of self-organization (and synergetics) as presented above. Therefore, just as in physics, the results presented above may serve as a basis for conducting laboratory experiments on bacteriophage infections to test the order parameter hypothesis. Moreover, we conclude that linear regression models as derived above may be used to estimate species that are difficult to observe on the basis of species that can be measured more conveniently. For sake of brevity, in the current study, properties of the endemic fixed point as studied, e.g., by Li et al. (2021) have not been examined in detail. Likewise, the current study was limited to consider one possible defense mechanism while alternative mechanisms (Skanata and Kussell, 2021; Weitz et al., 2005) were ignored. A more comprehensive study (which is beyond the scope of this Brief Report) may overcome such limitations by generalizing the results presented above.

## Data Availability

The raw data supporting the conclusions of this article will be made available by the authors, without undue reservation.
